# Plasmonic O_2_ dissociation and spillover expedite selective oxidation of primary C–H bonds†

**DOI:** 10.1039/d1sc04632b

**Published:** 2021-11-05

**Authors:** Hao Li, Huan Shang, Fuze Jiang, Xingzhong Zhu, Qifeng Ruan, Lizhi Zhang, Jing Wang

**Affiliations:** Institute of Environmental Engineering, ETH Zürich Zürich 8093 Switzerland jing.wang@ifu.baug.ethz.ch; Laboratory for Advanced Analytical Technologies, Empa, Swiss Federal Laboratories for Materials Science and Technology Dübendorf 8600 Switzerland; Laboratory of Pesticide & Chemical Biology of Ministry of Education, Institute of Applied & Environmental Chemistry, College of Chemistry, Central China Normal University Wuhan 430079 China; College of Science, Nanjing University of Aeronautics and Astronautics Nanjing 210016 China; Engineering Product Development, Singapore University of Technology and Design Singapore 487372 Singapore

## Abstract

Manipulating O_2_ activation *via* nanosynthetic chemistry is critical in many oxidation reactions central to environmental remediation and chemical synthesis. Based on a carefully designed plasmonic Ru/TiO_2−*x*_ catalyst, we first report a room-temperature O_2_ dissociation and spillover mechanism that expedites the “dream reaction” of selective primary C–H bond activation. Under visible light, surface plasmons excited in the negatively charged Ru nanoparticles decay into hot electrons, triggering spontaneous O_2_ dissociation to reactive atomic ˙O. Acceptor-like oxygen vacancies confined at the Ru–TiO_2_ interface free Ru from oxygen-poisoning by kinetically boosting the spillover of ˙O from Ru to TiO_2_. Evidenced by an exclusive isotopic O-transfer from ^18^O_2_ to oxygenated products, ˙O displays a synergistic action with native ˙O_2_^−^ on TiO_2_ that oxidizes toluene and related alkyl aromatics to aromatic acids with extremely high selectivity. We believe the intelligent catalyst design for desirable O_2_ activation will contribute viable routes for synthesizing industrially important organic compounds.

## Abstract 2

Room-temperature O_2_ dissociation and spillover, as driven by plasmonic Ru on oxygen-deficient TiO_2_, expedite the selective oxidation of primary C–H bonds in alkyl aromatics for synthesizing industrially important organic compounds.

## Graphical abstract

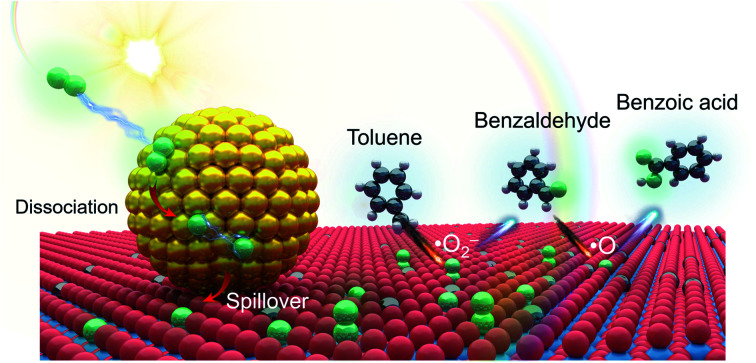

## Introduction

Our Earth's atmosphere is relatively rich in molecular oxygen (O_2_). This is attributed to the photosynthesis by cyanobacteria that led to the “great oxidation event” about 2.5 billion years ago. The high reduction potential of O_2_ makes it an excellent green oxidizing agent, while the triplet ground state of O_2_, with two unpaired electrons occupying two antibonding π orbitals in the same spin direction, represents a significant challenge for its robust utilization.^1^ To overcome this prominent difficulty, nature evolves families of metalloproteins, which contain unpaired d-electrons, to metabolize O_2_*via* stepwise reduction.^2^ In light of this, heterogeneous catalysts with redox transition metal centers were fabricated accordingly to mimic the functionality of enzymatic O_2_ utilization.^3,4^ The investigation of these processes is known collectively as O_2_ activation, aiming at integrating environmentally benign substances into global industrialization that encompasses a critical set of applications, including fuel cells, environmental remediation, and most importantly, fine chemical synthesis.^5^ Unfortunately, compared with metalloproteins, which can be operated under ambient conditions, artificial O_2_ activation still suffers from poor efficiency. For instance, the rates of selective (partial) oxidation reactions (ethylene epoxidation, alcohol transformation, and CH_4_ oxidation) are kinetically limited by the stubborn O–O double bond of O_2_ towards dissociation.^4,6–8^ To surmount the energy barrier, high operating temperatures are usually crucial if decent oxidation rates are to be expected. Paradoxically, thermal heating decreases energy efficiencies for numerous inherent exothermic oxidation reactions, compromises the long-term stability of catalysts, or gives rise to low product selectivity.^6,9–11^ To this end, developing alternative technologies that enable efficient O_2_ activation under ambient temperatures is currently an ambitious goal pursued by worldwide researchers.

Solar light is a clean and limitless energy source that can meet the world's energy needs. From a sustainable chemistry perspective, a conceptually promising approach for facile O_2_ activation is heterogeneous photocatalysis.^12–15^ By virtue of the photo-excited charge carriers, O_2_ can be easily activated to a series of reactive oxygen species (ROS) at room temperature, including ˙O_2_^−^, O_2_^2−^, ^1^O_2_, and ˙OH. However, one serious drawback of photocatalytic O_2_ activation is that ROS are usually randomly generated.^13,16^ Since different ROS show distinct redox chemistry (interactions) with a specific reactant, multiple thermodynamic reaction pathways, mediated by various co-existing ROS, can proceed simultaneously.^17,18^ Thus, without precise tuning of ROS, unwanted toxic intermediates or byproducts will emerge, giving rise to low selectivity for target molecules.^1,19^ In this context, manipulating photocatalytic O_2_ activation towards the generation of desirable ROS is intuitively valid and theoretically reliable for efficient and selective oxidation reactions. The traditional viewpoint on manipulating photocatalytic O_2_ activation is overwhelmingly focused on tuning the electronic structures of photocatalysts. On the surface molecular level, the collaborative development of modern material characterizations and computations, as motivated by nanosynthetic chemistry, highlights that delicate surface structures (exposed surface, native defects, and interfacial configurations) play a more pivotal role in defining the activation manner of small ambient molecules (H_2_O, CO_2_, N_2_).^13,20–22^ Therefore, the integration of surface science with photocatalytic O_2_ activation opens a new avenue to regulate the generation and transformation of desirable ROS in foreseen scenarios.

Here, we report a new O_2_ activation mechanism based on a carefully designed nanostructured Ru/TiO_2−*x*_ photocatalyst. The Ru nanocatalyst on the TiO_2−*x*_ (oxygen-deficient TiO_2_ substrate), in the negatively charged state, is highlighted here to trigger room-temperature O_2_ dissociation through surface plasmons. The acceptor-like oxygen vacancies confined at the interface kinetically boost ˙O diffusion and spillover from Ru to TiO_2_, thus avoiding oxygen-poisoning and catalyst deactivation. The spiltover O_2_, in the form of atomic ˙O, is highly active, and together with native ˙O_2_^−^ on TiO_2_, it can expedite the “dream reaction” of selective primary C–H bond activation. Evidenced by an exclusive isotopic ^18^O-transfer phenomenon, this novel photocatalyst oxidizes toluene into benzoic acid with selectivity over 97% under visible light. Mechanistic insights into the plasmonic O_2_ dissociation and spillover scheme, as well as the origination of the high selectivity, are comprehensively discussed on the basis of theoretical and experimental results. As a proof-of-concept, several other related alkyl aromatics are used to showcase the potential of Ru/TiO_2−*x*_ for extended applications.

## Results and discussion

### Theoretical scenarios of O_2_ adsorption, activation, and dissociation

**Fig. 1:**
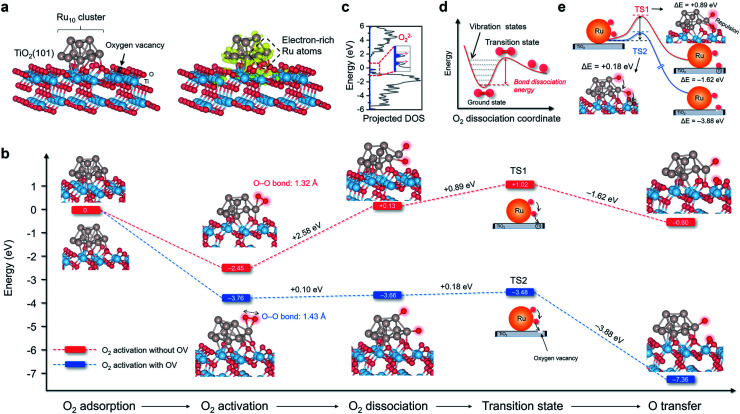
Thermodynamics and kinetics of O_2_ activation, dissociation, and spillover on Ru_10_ cluster/anatase(101) surface with and without OV. (a) The theoretical model of Ru/TiO_2−*x*_. The charge density localization on the Ru_10_ cluster is shown on the right side. The yellow isosurface with an isovalue of 0.005 au represents spatial charge accumulation. (b) Free energy change against the reaction coordinates for O_2_ activation, dissociation, and spillover over Ru/TiO_2_ with and without OV. (c) Molecular density of states projected on highly activated O_2_ adsorbed on Ru/TiO_2−*x*_. For clarity, the spin-down plots are not shown here. (d) Schematic illustration of the potential energy surface towards O_2_ dissociation. (e) Transition states associated with O atom spillover from Ru_10_ to TiO_2_ substrate with and without OV.

TiO_2_ is the most widely studied photocatalyst owing to its high abundance and chemical stability. However, O_2_ interacts weakly with the perfect TiO_2_ surface, impeding photoelectron-driven O_2_ activation. Thus, we sought to construct a multifunctional nanostructure to manipulate O_2_ activation using TiO_2_ as the primary building block.^23^ The first ingredient we considered was the oxygen vacancies (OVs), which are the most common anion defects to promote oxygen adsorption and diffusion.^24,25^ The second building block we thought of was Ru due to the following two reasons. First, Ru catalyst with d-band electrons shows a very high affinity to O_2_. It has been extensively employed for thermally driven catalytic oxidations, including CO, CH_4_, alcohol, acid, alkene, and biomass oxidations.^26^ Second, after being downsized to the nanoscale regime, nanosized Ru displays strong interactions with the oscillating electric field of the incident light, known as the localized surface plasmon resonance (LSPR) phenomenon.^27–30^ LSPR excitation is accompanied by the generation of abundant plasmons or hot electrons that may be energetic enough to activate O_2_ at room temperature. Density functional theory (DFT) calculation was then performed to depict the O_2_ adsorption, activation, and possible dissociation processes on Ru/TiO_2_ nanocomposites. Anatase(101) is one of the representative surfaces of TiO_2_ with high thermodynamic stability. We optimized the geometric structure of the anatase(101) surface with an OV on the O row (TiO_2−*x*_). A 10-atom Ru (Ru_10_) cluster with a hexagonal close-packed crystal structure, which usually represents nanoparticles, was placed on the bridging O row close to the OV on TiO_2−*x*_ to construct a Ru/TiO_2−*x*_ composite structure (Fig. 1a). According to the charge density difference, the Ru_10_ cluster became negatively charged (Fig. 1a). Bader charge analysis demonstrated that the electron-rich OV center at the interface transferred (donated) one of its localized electrons to Ru_10_. Those negatively charged Ru atoms close to the interfacial OV with low steric hindrance were deemed extremely active for O_2_ adsorption and activation. As expected, the O_2_ adsorption on negatively charged Ru_10_ was exergonic by 3.76 eV (Fig. 1b). Consistent with the enormous adsorption energy, the O–O bond of O_2_ was largely activated to 1.43 Å, close to that of O_2_^2−^ (1.48 Å). Based on the spin of adsorbed O_2_ that was composed of an occupied majority and minority state without any magnetic moment, the activated O_2_ on Ru/TiO_2−*x*_ was reconfirmed to be O_2_^2−^ species (Fig. 1c).^31^ In contrast, for the Ru_10_ cluster on defect-free TiO_2_ (Ru/TiO_2_), O_2_ adsorption was only exergonic by 2.45 eV with a slightly stretched O–O bond length of 1.32 Å (Fig. 1b). Clearly, with an OV confined at the Ru–TiO_2−*x*_ interface, both O_2_ adsorption and activation on Ru_10_ were remarkably strengthened. After identifying the O_2_ pre-activation state, we were curious about whether O_2_ dissociation would proceed further. For O_2_ on the ground state potential energy surface (PES), its dissociation is encountered by an intense energy barrier ascribed to the strong O–O double bond, suggesting that room-temperature O_2_ dissociation is energy demanding (Fig. 1d). Interestingly, with an OV at the Ru–TiO_2−*x*_ interface, O_2_ dissociation became thermodynamically favorable with a negligible energy barrier of +0.10 eV (Fig. 1b). For O_2_ on Ru/TiO_2_ in a much weaker activation state, its dissociation, by comparison, was strongly endergonic by +2.58 eV.

Following O_2_ dissociation, the next step we considered was the transportation of the dissociated O_2_. The ideal case is that part of the ˙O can be transferred to the TiO_2_ surface so that Ru is free from O_2_ poisoning and readily “pumps” ˙O to the TiO_2_ surface, where reactant adsorption takes place. Such a step can be denoted as O_2_ spillover, a general concept in thermocatalysis that depicts the migration of reactive species adsorbed on one surface to another surface that does not adsorb or generate the species directly under the same conditions.^32^ According to the transition state (TS), even though ˙O spillover from Ru_10_ to TiO_2_ was exergonic by 1.62 eV, the kinetic barrier was as large as +0.89 eV (Fig. 1b). Interestingly, ˙O could easily spillover to the OV confined at the Ru/TiO_2−*x*_ atomic interface with an energy release of 3.88 eV. Moreover, the kinetic barrier of ˙O spillover towards the OV was remarkably reduced to +0.18 eV (Fig. 1b). After a careful examination of the sophisticated TS structures, we found that ˙O spillover on Ru/TiO_2_ was restricted by the interfacial steric hindrance and the sizeable electrostatic repulsion with lattice O (Fig. 1e). In contrast, the OV confined at the Ru–TiO_2−*x*_ interface acted as a perfect oxygen acceptor and readily accommodated the spillover ˙O, which well explained the thermodynamic and kinetic feasibility of oxygen spillover (Fig. 1e).

### Synthesis of the Ru/TiO_2−*x*_ photocatalyst

**Fig. 2:**
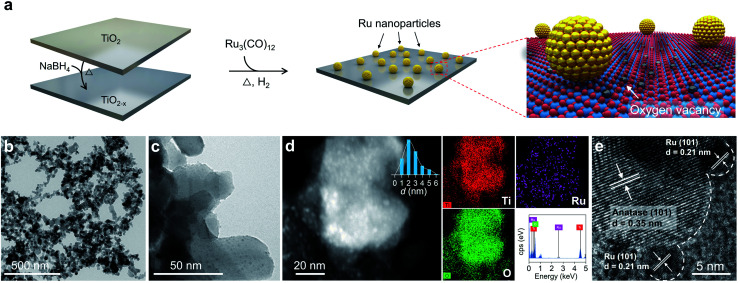
Preparation and electron microscopy characterization of the Ru/TiO_2−*x*_ photocatalyst. (a) Schematic illustration of the Ru/TiO_2−*x*_ preparation process. (b and c) TEM images of Ru/TiO_2−*x*_. (d) HAADF-STEM and EDX mapping images of Ru/TiO_2−*x*_. (e) HRTEM image of Ru/TiO_2−*x*_.

Enlightened by the theoretical results, both O_2_ dissociation and spillover on Ru/TiO_2−*x*_ were envisioned as highly practicable through acceptor-like OV under mild conditions. To verify our hypothesis, a Ru/TiO_2−*x*_ nanocomposite was prepared accordingly. The preparation process was associated with the preliminary reduction of commercial TiO_2_ by NaBH_4_ through calcination to obtain TiO_2_ with abundant surface OVs (TiO_2−*x*_). Then, Ru_3_(CO)_12_, impregnated on TiO_2−*x*_, was reduced by a gaseous mixture of H_2_ and Ar (1 : 9, v/v) at 350 °C to obtain Ru/TiO_2−*x*_ (Fig. 2a). For comparison, we synthesized Ru/TiO_2_ through the same H_2_-annealing method only by replacing TiO_2−*x*_ with defect-free TiO_2_. Transmission electron microscopy (TEM) showed that Ru/TiO_2−*x*_ consisted of nanoparticles with diameters ranging from 20 to 60 nm (Fig. 2b). Ru nanoparticles with an average size of 2 nm were highly dispersed on TiO_2−*x*_, which appeared brighter on the high-angular annular dark field-scanning transmission electron microscopy (HAADF-STEM) image due to the heavier atomic mass of Ru than that of Ti (Fig. 2c and d). Corresponding energy dispersive X-ray (EDX) mapping images showed that the support was composed of Ti and O, while the bright spots were made of Ru (Fig. 2d). The high-resolution TEM (HRTEM) image demonstrated the crystalline nature of native TiO_2−*x*_ and loaded Ru nanoparticles (Fig. 2e). The clear lattice fringes with a spacing of 0.35 nm and 0.21 nm corresponded to the anatase(101) and hexagonal Ru(101) atomic planes, respectively.

### Characterizations of the plasmonic Ru/TiO_2−*x*_ photocatalyst

**Fig. 3:**
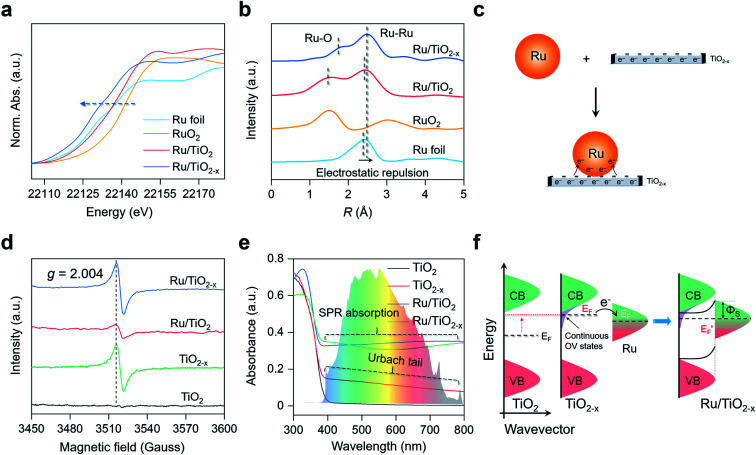
Characterization of the as-prepared TiO_2_, TiO_2−*x*_, Ru/TiO_2_, and Ru/TiO_2−*x*_. (a) XANES spectra, (b) Fourier transforms of Ru K-edge extended XAFS oscillations of the as-prepared photocatalysts. (c) Schematic illustration of the negatively charged Ru on TiO_2−*x*_. (d) EPR and (e) UV-vis absorption spectra of the as-prepared photocatalysts. (f) Schematic illustration of the interfacial charge transfer and Schottky barrier formation at the Ru–TiO_2−*x*_ interface.

X-ray diffraction (XRD) patterns revealed that the introduction of OVs and Ru nanoparticles did not change the crystal structure of TiO_2_, consistent with the TEM images (Fig. S1a†). No diffraction peaks assigned to Ru nanoparticles were detected in the XRD patterns of Ru/TiO_2_ or Ru/TiO_2−*x*_, possibly because the surface Ru content (∼2 atom%) was below the detection limit. The Ru K-edge X-ray absorption near-edge structure (XANES) spectra of Ru/TiO_2−*x*_ were significantly away from RuO_2_, but were slightly shifted to lower energies compared to that of Ru foil, indicating that the metallic Ru nanoparticles on TiO_2−*x*_ were negatively charged (Fig. 3a). We further adopted X-ray absorption fine structure (XAFS) to investigate the Ru atom relaxation behaviors. The Fourier transform of Ru K-edge extended XAFS oscillation curves of Ru/TiO_2_ and Ru/TiO_2−*x*_ were close to Ru foil but different from that of RuO_2_, reconfirming the metallic nature of the Ru nanoparticles (Fig. 3b). Interestingly, the Ru–Ru bond of Ru/TiO_2−*x*_ (∼2.5 Å) was notably longer than that of Ru/TiO_2_ or Ru foil (∼2.3 Å) based on the XAFS oscillation curves, suggesting the electrostatic repulsion within a negatively charged Ru nanoparticle (Fig. 3b). Consistent with the theoretical calculation, the OVs of TiO_2−*x*_ could donate part of their localized electrons, thus negatively charging the neighboring Ru nanoparticle and causing electrostatic repulsions (Fig. 3c).

Electron paramagnetic resonance (EPR) is a versatile technique to probe the type and concentration of defects. According to room-temperature EPR, TiO_2−*x*_ and Ru/TiO_2−*x*_ possessed a symmetrical and comparable signal with the specific *g* factor at 2.004 that corresponded to OVs (Fig. 3d). The OVs were also reflected by the two additional shoulder peaks with lower binding energies at 463.2 eV and 458.0 eV in the Ti 2p high-resolution X-ray photoelectron spectroscopy (XPS), assigned to the Ti^3+^ species around the OVs (Fig. S1b†). The concentration of OVs on TiO_2−*x*_ was quantitatively determined by the percentage of Ti^3+^ species, which was estimated to be 37% based on XPS analysis (Fig. S1b†). A weak OV signal appeared on Ru/TiO_2_ due to the H_2_-annealing process that slightly reduced the TiO_2_ substrate (Fig. 3d). Compared to white TiO_2_ with an absorption edge around 400 nm (bandgap ∼ 3.10 eV), grayish TiO_2−*x*_ displayed a decaying absorption tail throughout the visible light region, referred to as the Urbach tail (Fig. 3e). The formation of the Urbach tail was induced by the OV-associated electronic states beneath the conduction band (CB) edge.^33–35^ With a high concentration of OVs on the TiO_2_ surface, abundant localized states progressively became hybridized with the CB, giving rise to an exponentially decreased electronic state embedded within the bandgap of TiO_2_ (Fig. 3f). The continuous OV-induced states, which were usually 0–1.5 eV below the CB of TiO_2_, largely shifted the Fermi level (*E*_F_) above that of Ru.^36,37^ The upshifted Fermi level enabled localized electron transfer from the OVs to Ru nanoparticles *via* the interfacial Ru–O bonds until their Fermi levels were aligned at equilibrium, rationalizing the negatively charged Ru nanoparticles on TiO_2−*x*_ (Fig. 3c and S2†). Meanwhile, once the thermal equilibrium was reached, a Schottky barrier (*Φ*_S_) was established at the Ru–TiO_2−*x*_ interface. As evidenced by steady-state and time-resolved photoluminescence (PL) spectroscopy, both the OVs and Schottky barrier (*Φ*_S_) contributed to the rapid separation of charge carriers (Fig. S3 and Table S1†).^38,39^ The finite-difference time-domain (FDTD) simulations reveal that a Ru sphere (diameter of 2 nm) on TiO_2_ displays a plasmonic absorption ranging from 100 nm to 600 nm centered at 215 nm (Fig. S4†). The LSPR response of Ru on the TiO_2−*x*_ surface was a pronounced and extended absorption throughout the visible light spectrum (Fig. 3e). Unlike colloidal Au or Ag nanoparticles with representative plasmon resonant absorption peaks, the wide and flat absorption curve of Ru/TiO_2−*x*_ was probably due to the plasmon hybridization effect among nanoparticles in close proximity on defective substrates.^40^

### Study of O_2_ dissociation and spillover

**Fig. 4:**
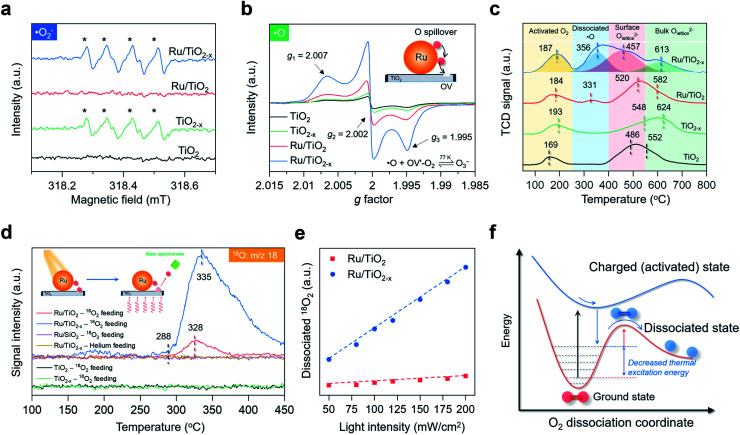
Investigation of O_2_ dissociation and spillover over the as-prepared photocatalysts. (a) Room-temperature EPR spectra of ˙O_2_^−^ in acetonitrile under visible light. (b) Low-temperature EPR spectra of O_3_^−^ under visible light. (c) O_2_-TPD spectra of the as-prepared photocatalysts. TCD represents the thermal conductivity detector. (d) O^18^ mass signals of the as-prepared photocatalysts after visible light irradiation. Inset represents a schematic illustration of O detection *via* TPD-MS. (e) The relative amount of dissociated ^18^O_2_ as a function of the light intensity. Dissociated ^18^O_2_ was calculated from the desorbed ^18^O-TPD-MS peak area. The dashed lines show the linear fit between temperature and dissociated ^18^O_2_. (f) Schematic illustration of plasmonic hot electron-driven O_2_ dissociation. The curves show the potential energy surface towards O_2_ dissociation.

After understanding the geometric and electronic structures of Ru/TiO_2−*x*_, we tried to detect the key ROSs formed at room temperature. According to theoretical calculation, the native ROS on the OV of anatase(101) surface was ˙O_2_^−^ (Fig. S5†). As evidenced by EPR, upon exposure to visible light, both TiO_2−*x*_ and Ru/TiO_2−*x*_ generated a four-line spectrum with relative intensities of 1 : 1 : 1 : 1 with 5,5-dimethyl-1-pyrroline-*N*-oxide as the spin-trapping reagent, a characteristic signal of ˙O_2_^−^ (Fig. 4a). The same signal was silent over Ru/TiO_2_, highlighting the pivotal role of OVs in enabling selective ˙O_2_^−^ formation (Fig. 4a). We did not detect any ˙OH signals due to the absence of water in acetonitrile (Fig. S6†). The direct detection of atomic ˙O in photocatalytic systems had been scarcely reported. We then sought to detect O_3_^−^ species, because if ˙O was spiltover to TiO_2−*x*_, it would probably interact with abundant native ˙O_2_^−^ to produce O_3_^−^ (˙O + O_2_^−^ → O_3_^−^). O_3_^−^ is described as the combination of ˙O and ˙O_2_^−^ through weak covalent bonding, which can be stabilized under low temperature and detected by EPR.^41–43^ In the air, irradiated Ru/TiO_2−*x*_ displayed three key parameters at *g*_1_ = 2.007, *g*_2_ = 2.002, and *g*_3_ = 1.995 at 77 K, a hyperfine structure corresponding to O_3_^−^ ions (Fig. 4b). Under the same conditions, neither TiO_2_ nor TiO_2−*x*_ afforded O_3_^−^ generation, suggesting that O_2_ dissociation was primarily initiated by plasmonic Ru nanoparticles. Meanwhile, the O_3_^−^-EPR spectra of Ru/TiO_2−*x*_ displayed two times stronger peak intensity than Ru/TiO_2_ (Fig. 4b). To directly validate plasmonic O_2_ spillover at room temperature, we performed O_2_-temperature-programmed desorption (O_2_-TPD) for the as-prepared photocatalysts after 30 min of visible light irradiation in the air. The O_2_-TPD profiles showed four types of oxygen species. The peaks at 100–250 °C, 250–400 °C, 400–550 °C, and 550–700 °C can be assigned to surface ˙O_2_^−^, atomic ˙O, lattice O on the surface, and lattice O in the bulk, respectively (Fig. 4c and S7†).^43^ In agreement with the low-temperature EPR, TiO_2_ and TiO_2−*x*_ showed negligible surface ˙O. However, O_2_-TPD of Ru/TiO_2−*x*_ exhibited a broad and intense desorption feature from 250 to 400 °C, indicating that spiltover ˙O from Ru was enriched on TiO_2−*x*_ (Fig. 4c). Based on the integrated O_2_-TPD peak area, the concentration of ˙O on Ru/TiO_2−*x*_ was 6 times that of Ru/TiO_2_, demonstrating that the O_2_ dissociation and spillover on Ru were kinetically promoted by OVs confined at the Ru–TiO_2−*x*_ interface (Fig. S7 and Table S2†).

Another direct evidence of room-temperature O_2_ dissociation and spillover was based on the TPD-mass spectrum (TPD-MS) (Fig. 4d). We deposited the Ru nanoparticles onto a SiO_2_ substrate (Ru/SiO_2_) for comparison (Fig. S8†). With He as the inert feeding gas, none of the photocatalysts exhibited O-desorption peaks after visible light irradiation (Fig. 4d). Interestingly, when ^18^O_2_ was used as the feeding oxygen, Ru/TiO_2−*x*_ showed a prominent peak at 335 °C with a mass fraction equal to ^18^O (Fig. 4d). The same ^18^O-desorption peak on Ru/TiO_2_ was at 328 °C, but much weaker. For TiO_2−*x*_ without Ru deposition, no mass signals of ^18^O were witnessed. In the dark, O_2_ dissociation was remarkably suppressed on Ru/TiO_2−*x*_ (Fig. S9†). Meanwhile, there was a linear relationship between the relative amount of dissociated ^18^O_2_ and light intensity, serving as the key signature of electron-mediated O_2_ dissociation (Fig. 4e).^6,44^ Both transient surface photovoltage and photocurrent response evidenced the enhanced formation of hot electrons on Ru nanoparticles when coupled with TiO_2−*x*_ (Fig. S10†). Contingent on the increased thermodynamic feasibility and boosted reaction kinetics, we reasoned that room-temperature O_2_ dissociation on Ru/TiO_2−*x*_ was primarily driven by plasmonic hot electrons. From the kinetic perspective, when surface plasmons of Ru are introduced as the external stimulus, hot electrons can transiently populate the antibonding orbital of O_2_ through plasmon decay and electron scattering that are highly dependent on the metal–O_2_ interplay.^29,30^ Thanks to the strong interactions between O_2_ and negatively charged Ru on TiO_2−*x*_, the dynamic plasmonic hot electron transfer from excited Ru to the antibonding π orbital of O_2_ will be kinetically boosted that quickly stretches the O–O bond of O_2_ to O_2_^2−^ species (O_2_ + 2e^−^ + *hν* → O_2_^2−^). In response to the antibonding orbital population, the nuclear motion along the O–O bond is promoted on the excited PES; still, the movement may not be so drastic to trigger direct O_2_ dissociation (overcome the O_2_ dissociation barrier) on excited-state PES due to the short lifetime of plasmonic electrons (Fig. 4f). Thus, an appreciable amount of plasmonic electrons decay back to the Ru, reverting O_2_^2−^ on the excited state PES to the ground state PES. During this decaying process, the plasmonic energy is not released but instead stored (deposited) in the O–O chemical bond (O_2_^2−^ − 2e^−^ → O_2_*), keeping O_2_ at a relatively high vibrational state with a much lowered thermal excitation energy barrier towards dynamic dissociation (Fig. 4f).^29,30,45^

### Selective primary C–H bond activation and mechanism

**Fig. 5:**
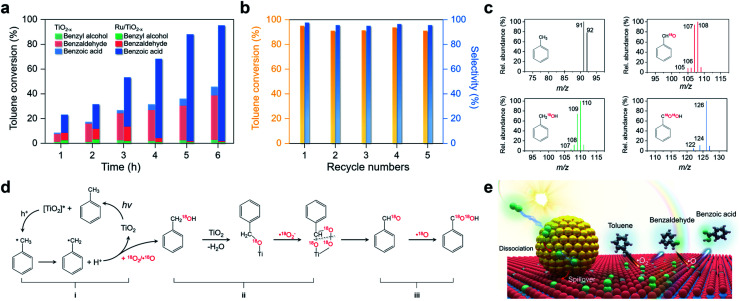
Selective photocatalytic oxidation of toluene and the proposed mechanism. (a) The temporal toluene conversion efficiency and selectivity over Ru/TiO_2−*x*_ and TiO_2−*x*_. (b) Multicycle selective toluene oxidation by Ru/TiO_2−*x*_. (c) Mass spectra of the oxygenated products with ^18^O_2_ as the oxidant generated by Ru/TiO_2−*x*_ after 3 hours of photoreaction. (d) Proposed mechanism for selective photocatalytic toluene oxidation to benzoic acid. (e) Schematic illustration of the plasmonic dissociation and spillover for selective toluene oxidation.

Activation of primary C–H bonds has long been the “dream reaction” to produce high-value-added chemicals from inexpensive raw chemicals. Unfortunately, traditional strategies with transition metal-complexes as the catalysts necessitate hazardous and refractory oxygen donors to drive the selective activation of the inert C(sp^3^)–H bonds under harsh conditions (*e.g.*, high pressure and high temperature with strong acidic or basic additives).^46–49^ Inspired by the novel plasmonic O_2_ dissociation and spillover phenomenon, we systematically evaluated the photoreactivity and selectivity of Ru/TiO_2−*x*_ for C–H bond activation at room temperature (26 °C). Using toluene, the simplest member of alkyl aromatics, as the model substrate, we found that TiO_2_, TiO_2−*x*,_ and Ru/TiO_2_ could successfully activate the primary C–H bonds of toluene under visible light. However, the conversion efficiencies remained low and the oxygenated product was a mixture of benzyl alcohol, benzaldehyde, and benzoic acid (Table 1). Subsequent separation and purification of the desired product add to the complexity of this process. Remarkably, Ru/TiO_2−*x*_ showed the highest toluene conversion efficiency (95.1%) with an impressive 97.1% selectivity towards benzoic acid (Table 1). Temporal evolution of intermediates and products showed that benzaldehyde and benzoic acid had been the dominant products of TiO_2−*x*_ (Fig. 5a). For Ru/TiO_2−*x*_, benzaldehyde and benzoic acid accounted for large fractions of oxidized toluene in the first 2 hours, whereafter benzoic acid gradually predominated (Fig. 5a). This provided direct evidence that toluene was oxidized to benzoic acid by Ru/TiO_2−*x*_ in a sequential manner. After another 4 cycles of photocatalytic toluene oxidation, Ru/TiO_2−*x*_ maintained its selectivity and reactivity (Fig. 5b). The XRD pattern, HRTEM image, and EPR spectra indicated that Ru/TiO_2−*x*_ was catalytically stable after multicycle photocatalytic toluene oxidation even though the concentration of OVs was slightly decreased (Fig. S11†). By comparison, Ru/SiO_2_ obtained a low toluene oxidation efficiency, consistent with the limited O_2_ dissociation capability on Ru/SiO_2_ (Table 1 and Fig. 4d). For a physical mixture of Ru nanoparticles and TiO_2−*x*_, its toluene oxidation was 52.5%, much lower than that of Ru/TiO_2−*x*_, highlighting the delicate Ru–TiO_2−*x*_ interactions in promoting photocatalytic toluene oxidation (Table S3†). It should be mentioned that the temperature of the solvent gradually increased from 25 to 41 °C without a cooling system with Ru/TiO_2−*x*_ as the photocatalyst under visible light. To rule out the contribution of increased temperature, we carried out toluene oxidation subject to a water bath heating that kept the temperature at 41 °C. Ru/TiO_2−*x*_ only obtained a 10.1% conversion efficiency in the dark at 41 °C (Table S3†). Increasing the sizes of Ru nanoparticles on TiO_2−*x*_ led to decreased efficiency for selective photocatalytic toluene oxidation, possibly due to occupied OVs, inhibited reactant adsorption, and decreased interfacial area (Fig. S12†).

**Table 1:** Photocatalytic oxidation of toluene by the as-prepared photocatalysts under visible light at room temperature (26 °C)^a^

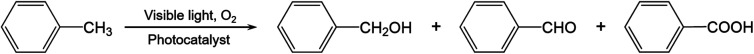					
Photocatalyst	Conversion (%)	Product selectivity (%)			
		Benzyl alcohol	Benzaldehyde	Benzoic acid	CO_2_
TiO_2_	18.8	9.7	50.3	38.2	1.8
TiO_2−*x*_	46.2	5.8	78.4	14.6	1.2
Ru/TiO_2_	26.3	3.8	29.1	65.6	1.5
Ru/TiO_2−*x*_	95.1	0.6	1.2	97.1	1.1
Ru/SiO_2_	18.4	10.8	47.8	39.8	1.6

a Reactions were carried out in 5 mL CH_3_CN solution, containing 0.1 mmol toluene and 50 mg photocatalyst at an O_2_ balloon pressure under a 300 W Xe lamp with a 400 nm cutoff filter. Acetonitrile was used as the solvent instead of water to avoid ˙OH generation. The distributions and concentrations of the products were determined by gas chromatography-mass spectrometry (GC-MS) at different reaction times.

To clarify the high selectivity of toluene oxidation towards benzoic acid, we focused on the contribution of different ROSs. When the reaction atmosphere was switched from O_2_ to Ar, the photo-conversion efficiency of Ru/TiO_2−*x*_ decreased dramatically to 13.9%, revealing that the primary oxidant was O_2_ (Fig. S13†). Then, different scavengers of reactive species were introduced into the reaction system. After AgNO_3_ was added to trap electrons, toluene conversion over TiO_2−*x*_ and Ru/TiO_2−*x*_ decreased significantly (Fig. S13†). As O_2_ alone could not oxidize toluene at room temperature, the electron-mediated O_2_ activation was considered vital for toluene oxidation. The addition of sodium oxalate (Na_2_C_2_O_4_) as the hole scavenger also suppressed toluene oxidation of TiO_2−*x*_ and Ru/TiO_2−*x*_ (Fig. S13†). The addition of benzoquinone as the ˙O_2_^−^ scavenger completely terminated toluene oxidation by TiO_2−*x*_. However, benzoquinone partially suppressed toluene oxidation by Ru/TiO_2−*x*_ accompanied by a decreased selectivity towards benzoic acid (Fig. S13 and Table S4†). This result suggested that besides ˙O_2_^−^, the contribution of another important ROS, presumably the ˙O, was indispensable to achieve a high benzoic acid selectivity. When tetra-methylpiperidine *N*-oxide (TEMPO) was added as a net scavenger for radical oxygen species (˙O_2_^−^ + ˙O), oxidation of toluene by Ru/TiO_2−*x*_ was inhibited entirely (Fig. S13†). Clearly, the spillover ˙O worked together with native ˙O_2_^−^ on TiO_2−*x*_ to expedite primary C–H bond activation of toluene for selective benzoic acid synthesis.

To unveil the selective toluene oxidation mechanism, we carried out ^18^O_2_ isotopic labeling experiments. After 2 hours of photoreaction, about 92% of benzyl alcohol and benzaldehyde molecules generated by Ru/TiO_2−*x*_ were ^18^O-labeled, ruling out the contribution of lattice O from TiO_2_ for toluene oxidation *via* the Mars–van Krevelen mechanism (Fig. 5c). After 6 hours of photoreaction, over 84% of the benzoic acid contained two ^18^O atoms (Fig. 5c). Meanwhile, the kinetic isotope effect (KIE) of O_2_ was 1.28 and 1.72 for TiO_2−*x*_ and Ru/TiO_2−*x*_, respectively (Fig. S14†). The KIE difference indicated that plasmonic O_2_ dissociation and spillover on Ru/TiO_2−*x*_ was more kinetically relevant to toluene oxidation than TiO_2−*x*_. Based on the results and discussion, we drew a plausible pathway for selective toluene oxidation by Ru/TiO_2−*x*_ (Fig. 5d). The first critical step was the activation of the primary C–H bonds in toluene by photoholes to form benzyl radical (i in Fig. 5d). The benzyl radical (carbon-centered radical) was evidenced by EPR with *n*-tertbutyl-α-phenylnitrone (PBN) as the spin-trapping reagent (Fig. S15†). Benzyl radicals then reacted with ^18^O_2_ or ˙^18^O to yield the ^18^O-labelled benzyl alcohol *via* an O-insertion reaction. Due to the selective production of ˙O_2_^−^ on native TiO_2−*x*_, benzyl alcohol was further oxidized to benzaldehyde through an O-exchange reaction between ˙O_2_^−^ and α-carbon of benzyl alcohol *via* an oxygen-bridged structure (ii in Fig. 5d).^17^ This step was verified by using ^16^O-benzyl alcohol as the substrate and ^18^O_2_ as the oxidant, which showed that ^18^O-labeled benzaldehyde emerged as the primary product on Ru/TiO_2−*x*_ (Fig. S16†). By increasing the concentration of OVs in Ru/TiO_2−*x*_, both photocatalytic toluene oxidation efficiency and selectivity were gradually increased (Fig. S17†). Since the Ru nanoparticles on Ru readily “pumped” ˙O onto the TiO_2−*x*_ surface through spillover, the major ROS responsible for the further oxidation of benzaldehyde into benzoic acid should be ˙^18^O (iii in Fig. 5d and e). This final step was further evidenced using ^16^O-benzaldehyde and ^18^O_2_ as the oxidant. TiO_2−*x*_ showed poor photoreactivity for benzaldehyde oxidation, while Ru/TiO_2−*x*_ selectively produced benzoic acid as the final product that contained one ^18^O atom (Fig. S18†). Overall, the selective activation of the primary C–H bonds in toluene resulted from the synergistic interaction between spiltover ˙O and native ˙O_2_^−^ (Fig. 5e). Ru/TiO_2−*x*_ was also active and selective for the oxidation of primary C–H bonds of a wide variety of substituted toluenes (Table S5†).

## Conclusions

In conclusion, guided by nanosynthetic chemistry, we first reported a room-temperature O_2_ dissociation and spillover mechanism that expedited the “dream reaction” of selective primary C–H bond activation with a plasmonic Ru/TiO_2−*x*_ catalyst. Under visible light, surface plasmons excited in negatively charged Ru nanoparticles decayed into hot electrons, triggering spontaneous O_2_ dissociation to reactive atomic ˙O. Acceptor-like oxygen vacancies confined at the Ru–TiO_2_ interface freed Ru from oxygen-poisoning by kinetically boosting the spillover of ˙O from Ru to TiO_2_. Evidenced by an exclusive isotopic O-transfer from ^18^O_2_ to oxygenated products, ˙O displayed a synergistic action with native ˙O_2_^−^ on TiO_2_ that oxidized toluene into benzoic acid with selectivity over 97%. The Ru/TiO_2−*x*_ was also active and selective for a number of other related alkyl aromatics with great potential for extended applications. We believe the intelligent photocatalyst design for desirable O_2_ activation will contribute viable routes for synthesizing industrially important organic compounds.

## Methods

### Catalyst preparation

To prepare TiO_2−*x*_, we thoroughly mixed 2 g commercial Degussa P25 TiO_2_ with 1 g NaBH_4_. The mixture was then transferred to a porcelain crucible with a cap and annealed at 300 °C (temperature increase rate: 10 °C min^−1^) in the air using a muffle furnace for 20 min. After naturally cooling down to room temperature, the grayish TiO_2−*x*_ was repeatedly washed with deionized water and ethanol 6 times to remove unreacted NaBH_4_, followed by vacuum drying at 80 °C. To prepare Ru/TiO_2−*x*_, 1 g of TiO_2−*x*_ was dispersed in tetrahydrofuran that contained Ru_3_(CO)_12_. The atomic percentage ratio of Ru to Ti was adjusted to 2 atom%. After 4 hours of impregnation in an Ar atmosphere, the black suspension was directly vacuum dried at 50 °C. Then, the black powder was reduced by a gaseous mixture of H_2_ and Ar (1 : 9, v/v) at 350 °C for 1 hour to obtain Ru/TiO_2−*x*_. For comparison, Ru/TiO_2_ and Ru/SiO_2_ were prepared through the same method only by replacing TiO_2−*x*_ with Degussa P25 and commercial SiO_2_.

### Photocatalytic toluene oxidation

In a typical process, photocatalytic toluene oxidation was carried out in 5 mL CH_3_CN that contained 0.1 mmol toluene. 50 mg photocatalyst was thoroughly immersed in the toluene solution and transferred to a 10 mL quartz tube. The CH_3_CN was first bubbled with O_2_ for 30 min to remove other dissolved gases completely. Then, the quartz tube was sealed with a balloon that was prefilled with O_2_ of high purity. Subsequently, the mixture was magnetically stirred for 1 hour in the dark in order to reach adsorption–desorption equilibrium. A water bath system was used to maintain the temperature of the quartz tube at around 26 °C. After light irradiation by a 300 W xenon lamp (Perfectlight: PLS-SXE 300) with a 400 nm cutoff filter for a certain time, the suspension was centrifuged and filtered with a nylon syringe filter (0.22 mm) to completely remove the nanoparticles. The oxygenated products in the solution were analyzed and determined with a gas chromatography-mass spectrometry instrument (Agilent Technologies, GC6890N, MS 5973, capillary column: HP-5MS, 30 m × 0.25 mm, 0.25 μm). The conversion efficiency of toluene and the selectivity for certain oxygenated products are defined as conversion (%) = [(*C*_i_ − *C*_T_)/*C*_i_] × 100% and selectivity (%) = [*C*_O_/(*C*_i_ − *C*_T_)] × 100%, where *C*_i_ is the initial concentration of toluene, *C*_T_ and *C*_O_ are respectively the concentrations of the detected toluene and corresponding oxygenated product. Typical ^18^O_2_-labeling photocatalytic experiments were carried out under the same conditions by replacing the O_2_ atmosphere with ^18^O_2_ (97 atom% ^18^O).

## Data availability

All experimental and theoretical supporting data are provided in the ESI.†

## Author contributions

H. L. and J. W. supervised the project. H. L. designed and carried out the experiments. H. S. and H. L. carried out the DFT calculations. X. Z. Z. and Q. F. R. carried out the FDTD simulation. F. Z. J. and L. Z. Z. contributed to the data analysis. H. L. and J. W. wrote the paper. All the authors discussed results and provided comments during the manuscript preparation.

## Conflicts of interest

There are no conflicts to declare.

## Supplementary Material

SC-012-D1SC04632B-s001
