# Supplementary material for: Plasmonic O2 dissociation and spillover expedite selective oxidation of primary C–H bonds
Source: Chem Sci. 2021 Nov 5;12(46):15308–17. doi: 10.1039/d1sc04632b (PMC8635223; doi:10.1039/d1sc04632b)
Supplement: SC-012-D1SC04632B-s001 [file SC-012-D1SC04632B-s001.pdf]

Electronic Supplementary Material (ESI) for Chemical Science.
This journal is © The Royal Society of Chemistry 2021

Supplementary Information for

Plasmonic O_2 Dissociation and Spillover Expedite Selective Oxidation of Primary C–H Bonds

Hao Li,[a,b] Huan Shang,[c] Fuze Jiang,[a,b] Xingzhong Zhu,[d] Qifeng Ruan,[e] Lizhi Zhang,[c] and Jing Wang*[a,b]

[a]Institute of Environmental Engineering, ETH Zürich, Zürich 8093, Switzerland

[b]Laboratory for Advanced Analytical Technologies, Empa, Swiss Federal Laboratories for Materials Science and Technology, Dübendorf 8600, Switzerland

[c]Laboratory of Pesticide & Chemical Biology of Ministry of Education, Institute of Applied & Environmental Chemistry, College of Chemistry, Central China Normal University, Wuhan 430079, China

[d]College of Science, Nanjing University of Aeronautics and Astronautics, Nanjing 210016, China

[e]Engineering Product Development, Singapore University of Technology and Design, Singapore 487372, Singapore

20 pages, 18 Figures, 5 tables

Supplementary Methods

Characterization methods. The powder X-ray diffraction (XRD) patterns of the samples were recorded on a Bruker D8 Advance diffractometer with monochromatized Cu Kα radiation (λ = 0.15418 nm). The morphology of the samples was observed with a JEOL 6700-F field-emission scanning electron microscope (SEM). The transmission electron microscopy (TEM) images were obtained by JEOL JSM-2010 high-resolution transmission electron microscopy. High-angular annular dark field-scanning transmission electron microscopy (STEM-HAADF) and energy dispersive X-ray (EDX) mapping images were obtained via JEOL, JEM-ARM200F. Chemical states were analyzed using X-ray photoelectron spectroscopy (XPS) (Thermo Scientific ESCLAB 250Xi). All binding energies were referenced to the C 1s peak (284.6 eV) arising from the adventitious carbon. UV-visible diffused reflectance spectra of the samples were obtained for the dry-pressed film samples with using a UV-visible spectrophotometer (UV-2550, Shimadzu, Japan) with $BaSO_4$ as the reflectance standard. Electron paramagnetic resonance (EPR) spectra were conducted on a Bruker EMX EPR Spectrometer (Billerica, MA). Steady-state and time-resolved photoluminescence (PL) measurements were carried out on an FLS900 fluorometer (Edinburgh Instruments). The X-ray absorption near-edge structure (XANES) spectra and extended X-ray absorption fine structure (EXAFS) were performed at the beamline 1W1B of Beijing Synchrotron Radiation Facility, Institute of High Energy Physics, Chinese Academy of Sciences. O_2-temperature-programmed desorption experiments (TPD) was carried out by AutoChem II 2920 (Micromeritics, USA). In a typical process, all the samples were pre-treated by high-purity He to remove adsorbed oxygen species. The system for steady-state and transient surface photovoltage (SPV) measurements included a source of monochromatic light, a lock-in amplifier (SR830-DSP) with a light chopper (SR540), a photovoltaic cell, and a computer. The samples were examined without further treatment during the SPV measurements, and the contact between the sample and the ITO electrode was non-ohmic.

First-Principles Density of Functional Theory (DFT) Calculation. All calculations were obtained by the density of functional theory (DFT) + *U*, utilizing the Vienna Ab-initio Simulation Package (VASP), in which the exchange-correlation energy functional was described by GGA-PBE.[1,2] The plane wave basis with 400 eV energy cutoff was employed for the valence electronic states expansion which was conducted by the projector augmented-wave (PAW) method.[3,4] The Hubbard *U* for Ti was 3 eV.[5,6] The k-points for Brillouin zone was 3 × 3 × 1. The vacuum was kept at 20 Å for all the models. The force and energy of the geometrical optimization were converged to 0.02 eV/Å and 10^{-5} eV/atom respectively. Location of

the transition states (TS) was determined by nudged elastic band (NEB) method.[7] Molecular O_2 adsorption was evaluated by adsorption energy, which was described by ΔE_{ad} (ΔE_{ad} = E(catalyst-O_2) - E(catalyst) - E(O_2)).

Finite-difference-time-domain (FDTD) simulation. FDTD simulation were performed using FDTD Solution 8.24 (Lumerical Solution). During the simulation, an electromagnetic pulse in the spectral range from 20 nm to 1100 nm was launched into a box containing the target nanostructure. A mesh size of 0.1 nm was employed in calculating the extinction spectra and charge distribution contours of the Ru nanosphere and Ru nanosphere-TiO_2. The refractive index of the surrounding medium was 1.33 of water. The dielectric function of Ru and TiO_2 were extracted from the D. W. Lynch et al. and M. W. Ribarsky et al., respectively.[8,9] The diameter of the Ru sphere was set to be 2 nm. The size of the TiO_2 was set as 5×5×2 nm. The Ru sphere with diameter of 2 nm were set on the middle surface of TiO_2 without gap.

Supplementary Figures

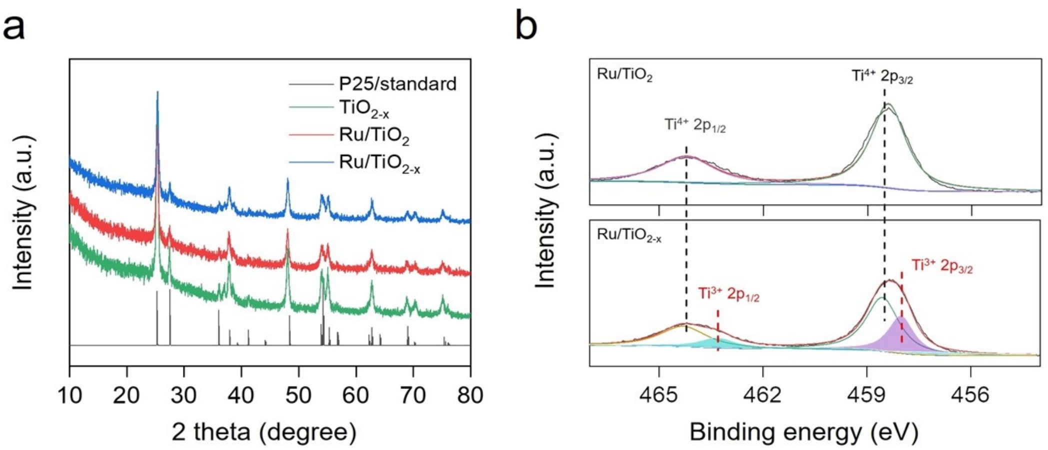

Fig. S1 (a) XRD pattern and (b) XPS spectra of the as-prepared photocatalysts.

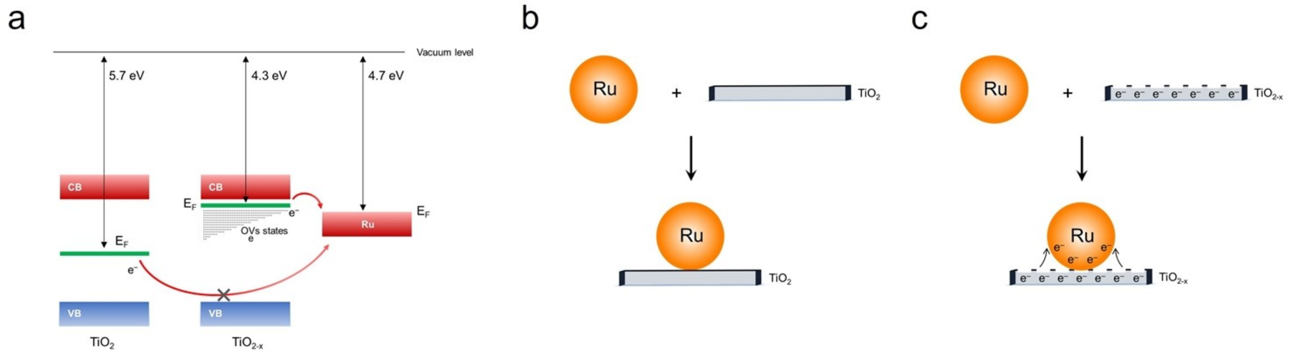

Fig. S2 (a) Schematic illustration of the band energy alignment and Fermi level (E_F) of Ru, TiO_2 and TiO_{2-x}. Schematic illustration of the interfacial charge transfer of (b) Ru/TiO_2 and (c) Ru/TiO_{2-x}.

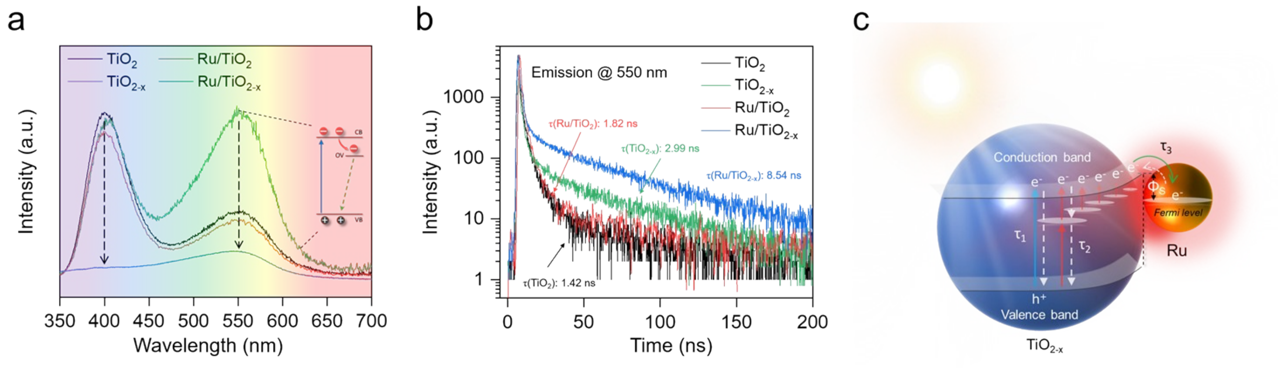

Fig. S3 (a) Steady-state and (b) time-resolved PL spectra (@550 nm) of the as-prepared photocatalysts. (c) Schematic illustration of charge carrier kinetics at the Ru-TiO_{2-x} interface. Both OVs and the Schottky barrier within Ru/TiO_{2-x} would affect the generation and transportation of charge carriers that determined the efficiency of photocatalysis.

To directly characterize charge carrier dynamics, informative photoluminescence (PL) spectroscopy was adopted. Under the excitation of a 300 nm laser, steady-state PL of TiO_2 displayed two emission peaks (Fig. S3a). The dominant peak at around 400 nm was the band-edge emission, while the other weaker one centered at 550 nm was from the radiative recombination of electrons trapped at OV states (~0.85 eV below the CB edge) with the holes at VB (inset of Fig. S3a).[10] Due to the high concentration of surface OVs on TiO_{2-x}, the defect emission peak became comparable to the band-edge emission. After Ru deposition, PL intensity of Ru/TiO_2 experienced no remarkable change, while both the band-edge and OV-induced emissions of Ru/TiO_{2-x} suffered from a substantial quenching (Fig. S3a). The PL quenching in Ru/TiO_{2-x} possibly stemmed from a combined effect between OVs and Schottky barrier on suppressing electron-hole recombination. To validate this assumption, we performed time-resolved PL measurements on the OV emission peak. According to the decay curves, both TiO_2 and TiO_{2-x} could be fitted with a double-exponential function, including a fast interband recombination decay component (τ_1) and a slow OV-mediated recombination decay component (τ_2) (Fig. S3b). Due to the efficient trapping of photoelectrons by OVs, TiO_{2-x} displayed a much higher proportion of the long-living component (τ_2, 68%), whose average time (2.99 ns) was therefore much longer than that of TiO_2 (1.42 ns) (Table S1). Ru/TiO_2 displayed an almost identical decay behavior to TiO_2, suggesting the weak interactions between Ru and TiO_2. As for Ru/TiO_{2-x}, its decay was the slowest among the as-prepared photocatalysts (Fig. S3b). After careful fitting, we found a third exponential constant (τ_3) with the longest lifetime of 19.05 ns was necessary to obtain a satisfactory fit, which further lengthened the carrier lifetime to 8.54 ns (Table S1). It is to be noted that the introduction of τ_3 was essential that signified the contribution of Schottky barrier. As schematically illustrated in Fig. S3c, the energetic electrons, either on the CB or trapped on the OV states, could be transferred to Ru nanoparticles. Whereas the presence of Schottky barrier at the interface would block the back-transfer of photoelectrons, thus suppressing their direct or indirect recombination with holes (Fig. S3c).

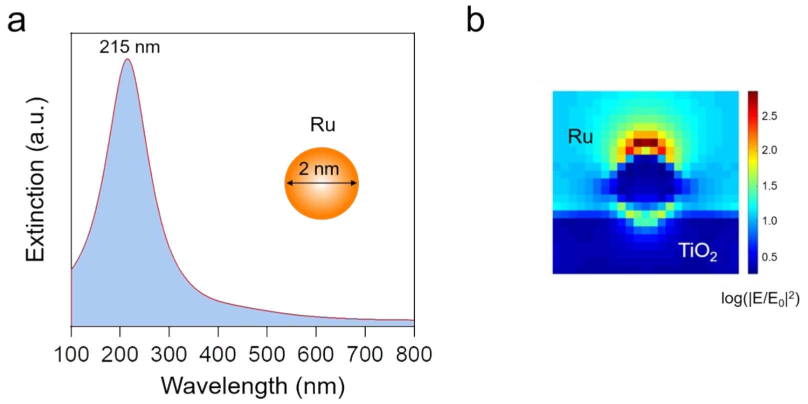

Fig. S4 Simulated extinction spectra of a Ru nanosphere of 2 nm in diameter supported on a TiO_2 nanosheet of 2 nm in thickness. Inset shows the electric field intensity enhancement contours of the Ru/TiO_2 hybrid system.

The finite-difference time-domain (FDTD) simulations reveal that a Ru sphere (diameter of 2 nm) on TiO_2 displays a surface plasmon resonance (SPR) absorption ranging from 100 nm to 600 nm centered at 215 nm (Fig. S4a).[11] It is to be noted that our experimentally determined UV-vis absorption spectrum displays a flat and extended absorption tail in the visible light region.[12] This enhanced and widened absorption tail could be possibly due to the hybridization effect between the defective substrate (TiO_{2-x}) and plasmonic metal (Ru), and also the hybridization among Ru nanoparticles.[13–15] Under visible light (λ = 420 nm), Ru can generate electric field intensity enhancement several times of the incident field ($|E/E_0|^2$) at the Ru-TiO_2 interface (Fig. S4b).

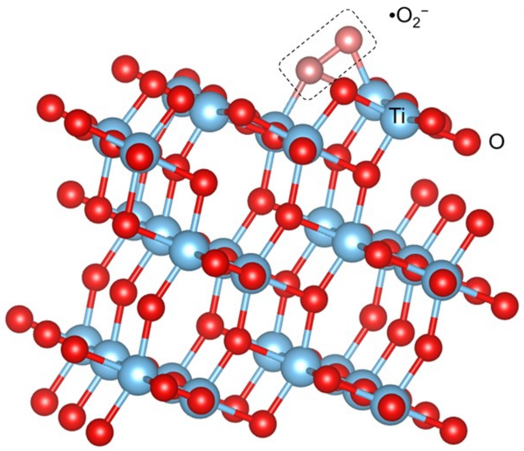

Fig. S5 Theoretical modeling of O_2 adsorption on the OV of anatase(101) surface.

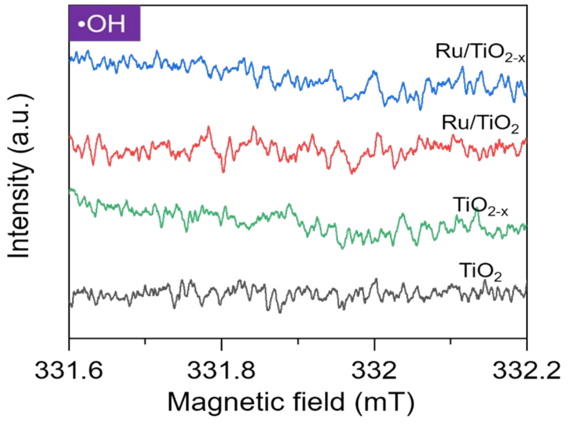

Fig. S6 Room-temperature EPR spectra of •OH in acetonitrile.

The product in Fig. S6 is a 5,5-dimethyl-1-pyrroline-*N*-oxide (DMPO) spin-trapping-•OH adduct, which is characterized by a four-line spectrum with relative intensities of 1 : 2 : 2 : 1. Since acetonitrile was used as the solvent, the possible generation of •OH via photocatalytic water oxidation (hole + H_2O → •OH) was ruled out.

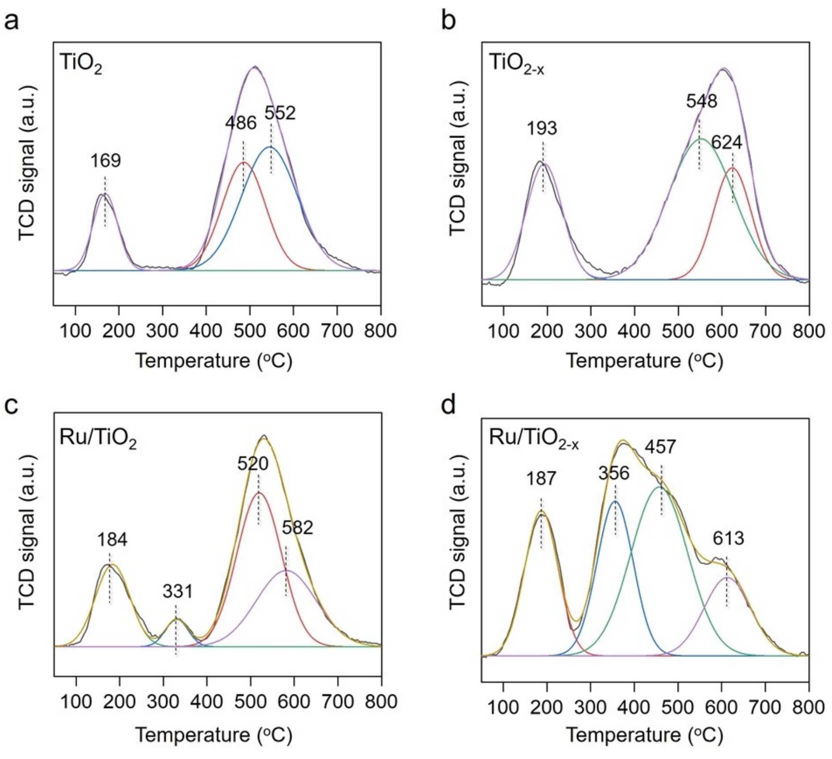

Fig. S7 Integration of the O_2-TPD peaks of the as-prepared photocatalysts.

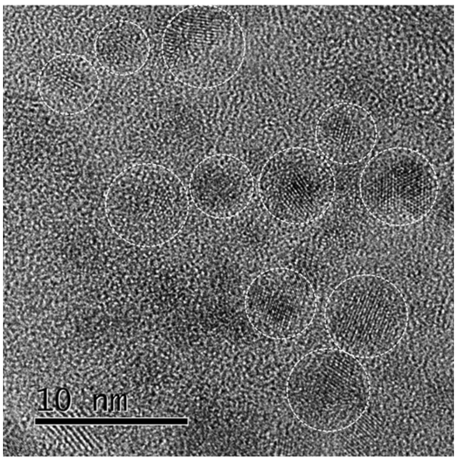

Fig. S8 HRTEM image of Ru/SiO_2 which shows the deposition of crystalline Ru nanoparticles on the amorphous SiO_2 substrate.

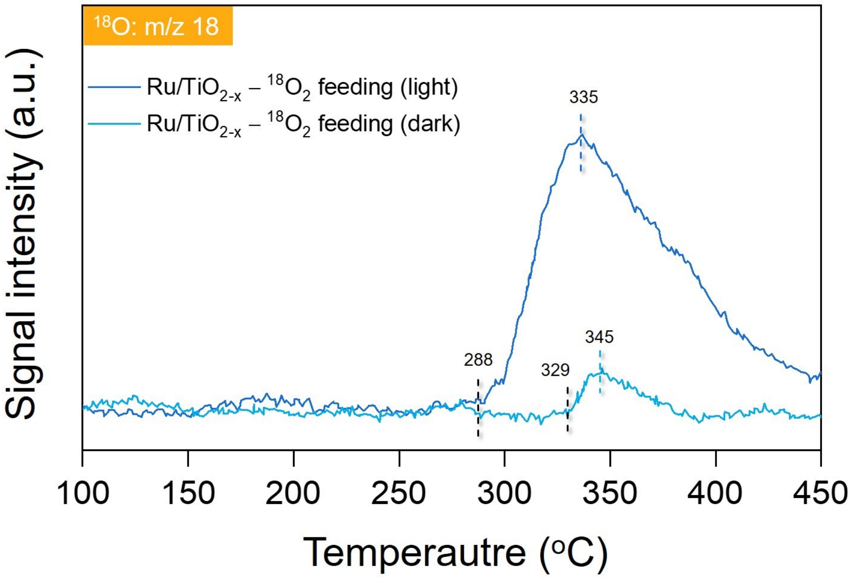

Fig. S9 •O^{18} mass signals of the as-prepared Ru/TiO_{2-x} with and without visible light irradiation.

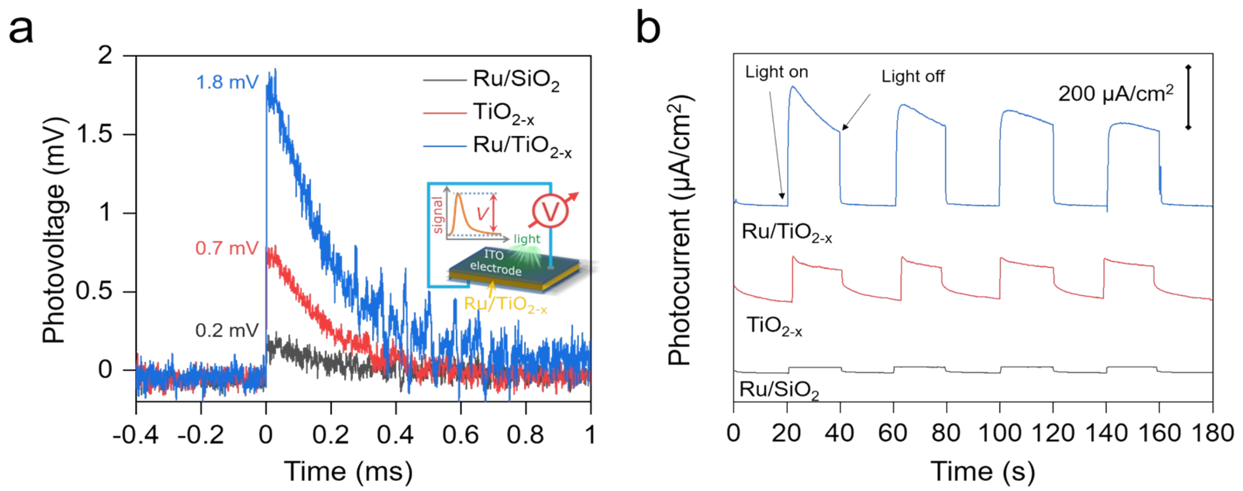

Fig. S10 (a) Transient SPV spectra of the photocatalysts under a 420 nm laser pulse. (b) Transient photocurrent response of the photocatalysts under visible light in an electrochemical system.

We first adopted transient surface photovoltage to probe the possible generation of hot electrons on Ru nanoparticles with a 420 nm laser pulse. Compared with TiO_{2-x} and Ru/SiO_2, Ru/TiO_{2-x} displays strongest transient photovoltage and longest lifetime (Fig. S10a), which is due to the formation of hot electrons on Ru nanoparticles when coupled to defective TiO_{2-x}.[16,17]

Photocurrent measurement was conducted on CHI660D Instruments in a standard three-electrode system with the as-prepared photocatalyst as the working electrode, Pt foil as the counter electrode, saturated calomel electrode as the reference electrode and 0.5 mol/L Na_2SO_4 aqueous solution as the electrolyte. Before the photocurrent measurement, Ar gas was purged into the Na_2SO_4 aqueous solution to remove the dissolved molecular oxygen for 30 min and kept purging during the photocurrent measurement. A LED light (UVEC-4II, Shenzhen Lamplic Technology Co. Ltd. China) with light intensity of 2 W/cm^2 was utilized as a monochromatic light source (λ = 420 nm) under a bias of +0.2 V. Similar to the surface photovoltage response, Ru/TiO_{2-x} displays the strongest photocurrent response compared with TiO_{2-x} and Ru/SiO_2, suggesting the generation of hot electrons by Ru nanoparticles on TiO_{2-x} (Fig. S10b).

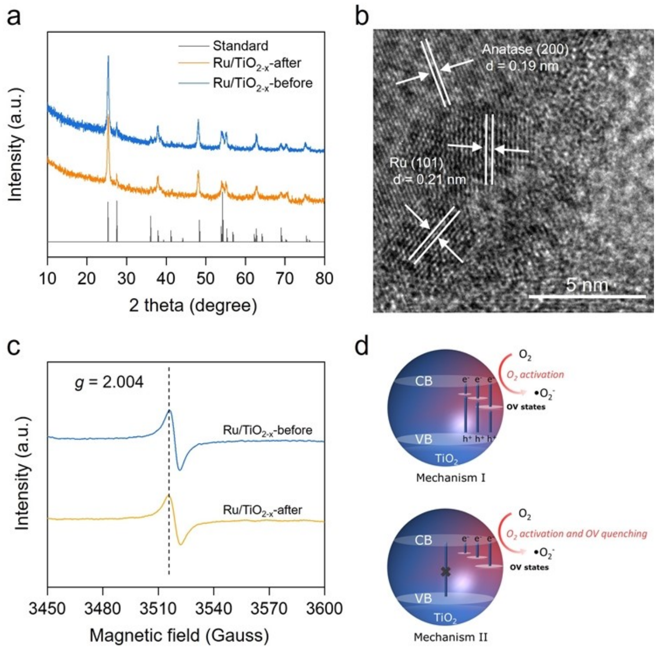

Fig. S11 (a) XRD patterns of Ru/TiO_{2-x} before and after multicycle photocatalytic toluene oxidation. (b) HRTEM image of Ru/TiO_{2-x} after multicycle test. (c) EPR spectra of Ru/TiO_{2-x} before and after multicycle test. (d) Schematic illustration of photocatalytic excitation in TiO_{2-x}.

XRD patterns of the Ru/TiO_{2-x} before and after multicycle photocatalytic toluene oxidation were the same, indicating Ru/TiO_{2-x} possessed good chemical stability for photocatalytic toluene oxidation (Fig. S11a). Meanwhile, after multicycle test, TEM image showed Ru nanoparticles on TiO_{2-x} were still in the metallic state based on the Ru(101) lattice fringes (Fig. S11b). Thus, the possible decomposition of TiO_{2-x} support and oxidation of Ru nanoparticles were ruled out.

It is highly possible that OVs in Ru/TiO_{2-x} were gradually oxidized during photocatalytic toluene oxidation. Based on the EPR spectra, OVs in Ru/TiO_{2-x} after multicycle test were slightly decreased (Fig. S11c). There are two mechanisms associated with the excitation of OVs in TiO_2. First, photoelectrons can be excited from the valence band of TiO_2 to the OVs states, and then to the conduction band (Fig S11d: Mechanism I). Second, the localized electrons on the OVs states can be directly excited to the conduction band of TiO_2 (Fig S11d: Mechanism II). After the localized electrons are trapped by adsorbed O_2, the OVs are quenched through the second mechanism. In our case, we believe both mechanisms co-exist. However, the dominant excitation pathway is inferred to be the first one, based on the slightly decreased OVs in Ru/TiO_{2-x} after multiple photocatalytic toluene oxidation.

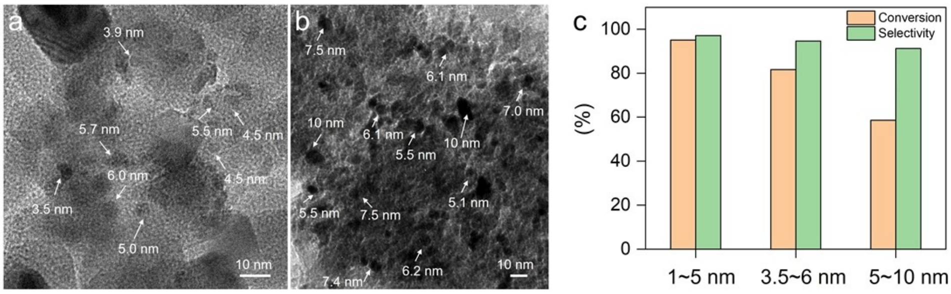

Fig. S12 Preparation of larger Ru nanoparticles on TiO_{2-x} with size of (a) 3.5~6.0 nm, and (b) 5.0~10 nm. (c) Effect of the Ru particle size on the conversion efficiency and selectivity for toluene oxidation into benzoic acid.

We prepared Ru nanoparticles with larger diameters from 3.5 nm to 6 nm, and from 5.0 nm to 10.0 nm by increasing the thermal reduction time of $Ru_3(CO)_{12}$ during the Ru/TiO_{2-x} synthesis (Fig. S12a and S12b). Increasing the Ru nanoparticle size led to the decrease of photocatalytic toluene oxidation activity (Fig. S12c). We reckon that larger Ru nanoparticle size might occupy the OVs, inhibit reactants adsorption, and decreases the interfacial area, all of which are disadvantageous for photocatalytic toluene oxidation.

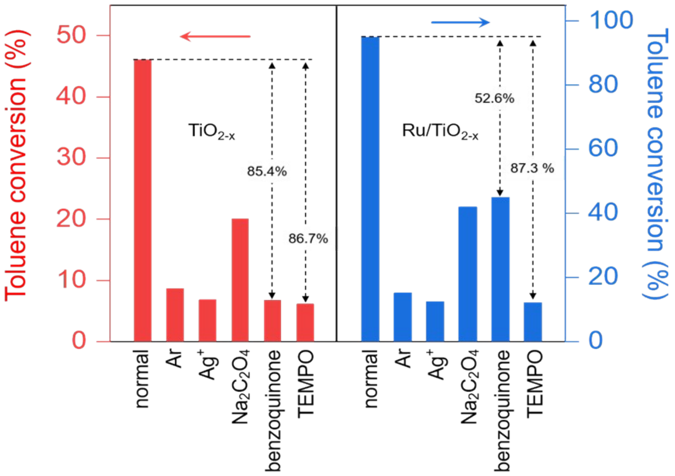

Fig. S13 Photocatalytic toluene conversion over TiO_{2-x} (left) and Ru/TiO_{2-x} (right) in the different environments or in the presence of the different trapping species. The concentration of the scavenger is 10 mM. Ag^+ is from the addition of $AgNO_3$. $Na_2C_2O_4$ and TEMPO represent sodium oxalate and tetra-methylpiperidine N-oxide, respectively.

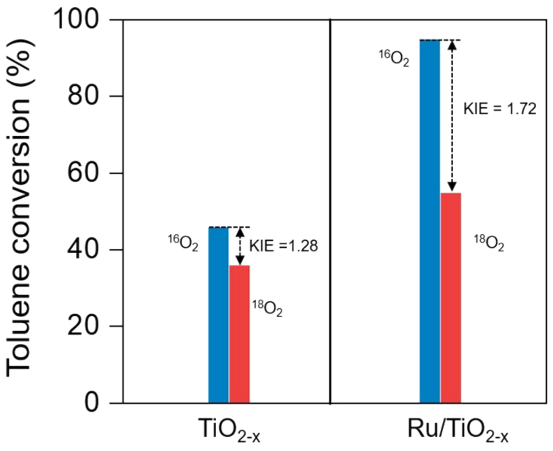

Fig. S14 Photocatalytic toluene conversion with $^{16}O_2$ and $^{18}O_2$ as the reactant over TiO_{2-x} (left) and Ru/TiO_{2-x} (right).

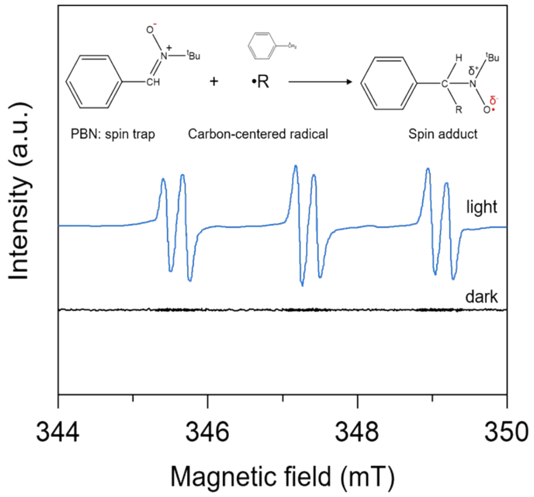

Fig. S15 Room-temperature EPR spectra of carbon centered radical using N-tert-butyl-α-phenylnitrone (PBN) as the spin-trapping reagent with Ru/TiO_{2-x} as the catalyst. The inset shows the spin trap reaction of carbon-centred radical with PBN.

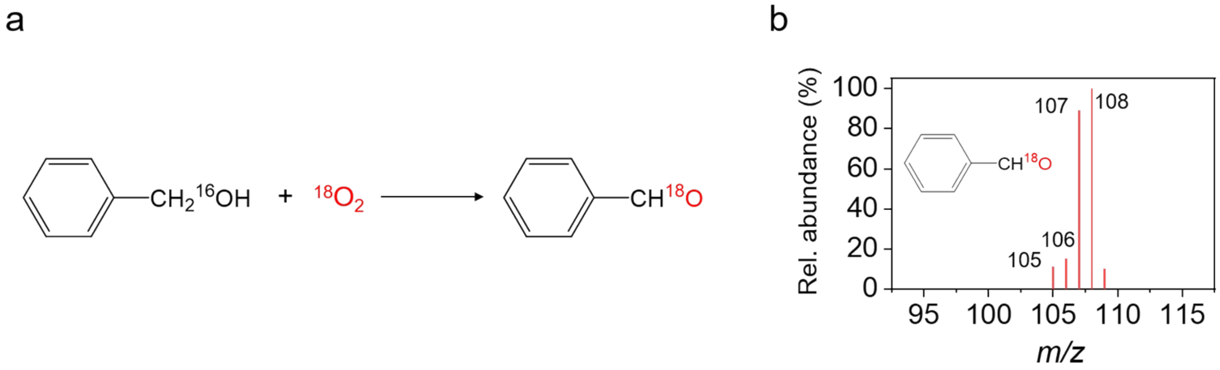

Fig. S16 (a) Photocatalytic oxidation of benzyl alcohol with $^{18}O_2$ as the reactant and Ru/TiO_{2-x} as the catalyst. (b) Mass spectra of corresponding benzaldehyde. Reactions were carried out in 5 ml CH_3CN solution, containing 0.1 mmol benzyl alcohol and 50 mg photocatalyst in 1 MPa $^{18}O_2$ under a 300-W Xe lamp with a 400 nm cutoff filter. After 4 hours of reaction, the products distributions and concentrations were determined by GC-MS.

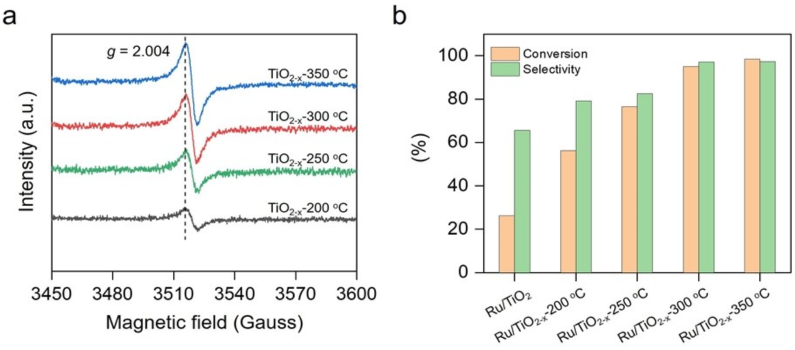

Fig. S17 (a) EPR spectra of TiO_{2-x} prepared under different annealing temperatures. (b) Influence of OVs (annealing temperature) in Ru/TiO_{2-x} on the conversion and selectivity for photocatalytic toluene oxidation.

To investigate the influence of OVs in Ru/TiO_{2-x} for photocatalytic toluene oxidation, we adjusted the annealing temperature for TiO_{2-x} synthesis from 200 °C to 350 °C with $NaBH_4$ as a reducing reagent. In our study, the default TiO_{2-x} used to synthesize Ru/TiO_{2-x} was prepared at 300 °C. According to the EPR spectra, the symmetrical signal of OV was gradually enhanced along with the annealing temperature increase (Fig. S17a). Subsequently, Ru nanoparticles were deposited onto TiO_{2-x} with different concentrations of OVs through the same method. It was interesting to find along with OVs increase, Ru/TiO_{2-x} displayed both enhanced conversion efficiency and selectivity for toluene oxidation (Fig. S17b). As discussed in our manuscript, the OVs on TiO_2 play an important role in facilitating O_2 activation into $\bullet O_2^-$ that

selectively oxidizes toluene into benzaldehyde. Together with the •O_2 dissociated from the Ru nanoparticles, benzaldehyde can be further oxidized into benzoic acid (Fig. 5d).

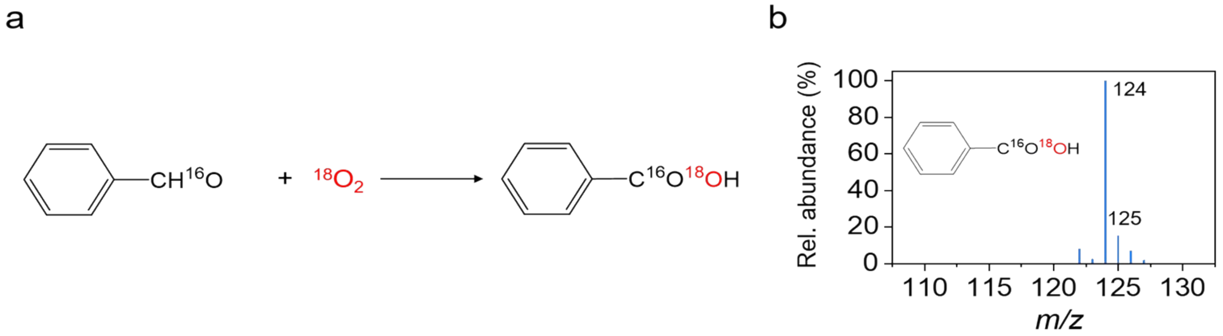

Fig. S18. (a) Photocatalytic oxidation of benzaldehyde with $^{18}O_2$ as the reactant and Ru/TiO_{2-x} as the catalyst. (b) Mass spectra of corresponding benzoic acid. Reactions were carried out in 5 ml CH_3CN solution, containing 0.1 mmol benaldehyde and 50 mg photocatalyst in 1 MPa $^{18}O_2$ under a 300-W Xe lamp with a 400 nm cutoff filter. After 4 hours of reaction, the products distributions and concentrations were determined by GC-MS.

Supplementary tables

Table S1. Parameters of the time-resolved PL decay curves of the as-prepared photocatalysts*

photocatalyst	decay time (ns)			relative percentage (%)			average lifetime (ns)	R^2
	τ_1	τ_2	τ_3	B_1	B_2	B_3		
TiO_2	0.72	3.53	-	75	25	-	1.42	0.95
TiO_{2-x}	0.81	4.02	-	32	68	-	2.99	0.98
Ru/TiO_2	0.68	3.86	-	64	36		1.82	0.96
Ru/TiO_{2-x}	0.75	3.18	19.05	21	42	37	8.54	0.93

*The decaying curve can be fitted using a multiexponential function $I_{PL}(t) = \sum_{i=1}^{n} A_i e^{t/\tau_i}$, where $I_{PL}(t)$ represents the PL intensity, τ_i the decay time, and A_i the amplitude. Average lifetime <τ> is calculated by $<\tau>= \sum_{i=1}^{n} B_i \tau_i$, where c_i is the relative concentration in the multiexponential decay and is expressed as $B_i = A_n \Big/ \sum_{i=1}^{n} A_i$.

Table S2. The integrated O_2-TPD peak area

catalysts	relative percentage (%)				
	activated O_2 (50-250 ^oC)	atomic •O (250-400 ^oC)	surface $O_{lattice}$ (400-550 ^oC)	bulk $O_{lattice}$ (550-800 ^oC)	R^2
TiO_2	14	-	35	51	0.997
TiO_{2-x}	24	-	52	24	0.993
Ru/TiO_2	19	**4**	46	31	0.997
Ru/TiO_{2-x}	20	**24**	40	16	0.997

Table S3. Photocatalytic oxidation of toluene with or without visible light irradiation*

entry	catalyst	light	conversion (%)
1	Ru/TiO_{2-x}	visible	95.1
2	physical mixture of the Ru nanoparticles with TiO_{2-x}	visible	52.5
3	Ru/TiO_{2-x}	no (at 41 ^oC)	10.1

*Reactions were carried out in 5 ml CH_3CN solution, containing 0.1 mmol toluene and 50 mg photocatalyst in 1 MPa O_2. After 6 hours of reaction, the products distributions and concentrations were determined by GC-MS.

Table S4. Photocatalytic oxidation of toluene by Ru/TiO_{2-x} with and without benzoquinone*

Entry	photocatalyst	conversion (%)	benzoquinone	product selectivity (%)		
				benzyl alcohol	benzaldehyde	benzoic acid
1	Ru/TiO_{2-x}	95.1	no	0.6	1.2	97.1
2	Ru/TiO_{2-x}	45.5	yes	5.8	31	61.4

*Reactions were carried out in 5 ml CH_3CN solution, containing 0.1 mmol toluene and 50 mg photocatalyst in 1 MPa O_2 under a 300-W Xe lamp with a 400 nm cutoff filter. After 6 hours of reaction, the products distributions and concentrations were determined by GC-MS.

Table S5. Photocatalytic oxidation of substituted toluenes towards corresponding acids by Ru/TiO_{2-x} under visible light*

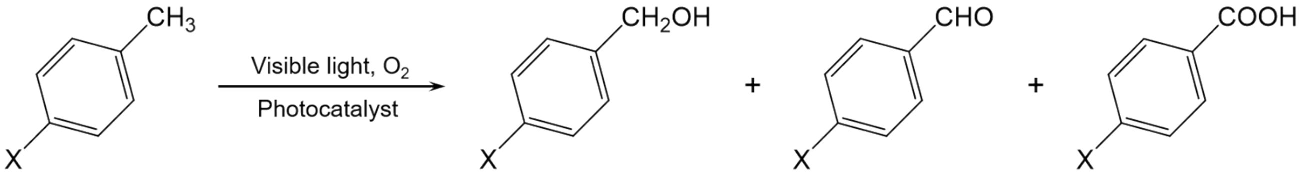

entry	–X	time (h)	conversion (%)	selectivity (%)
1	–H	6	95.3	97.1
2	–OCH_3	6	94.8	95.7
3	–CH_3	6	96.1	95.5
4	–F	6	88.5	92.8
5	–NO_2	6	82.6	93.3

*Reactions were carried out in 5 ml CH_3CN solution, containing 0.1 mmol substrate and 50 mg photocatalyst in 1 MPa O_2 under a 300-W Xe lamp with a 400 nm cutoff filter. After 6 hours of reaction, the products distributions and concentrations were determined by GC-MS.

Supplemental References

1 J. P. Perdew, K. Burke and M. Ernzerhof, *Phys. Rev. Lett.*, 1996, **77**, 3865–3868.

2 S. L. Dudarev, G. A. Botton, S. Y. Savrasov, C. J. Humphreys and A. P. Sutton, *Phys. Rev. B*, 1998, **57**, 1505–1509.

3 G. Kresse, *Phys. Rev. B*, 1996, **54**, 11169–11186.

4 G. Kresse and D. Joubert, *Phys. Rev. B*, 1999, **59**, 1758–1775.

5 M. Setvín, U. Aschauer, P. Scheiber, Y.-F. Li, W. Hou, M. Schmid, A. Selloni and U. Diebold, *Science*, 2013, **341**, 988–91.

6 A. Tilocca and A. Selloni, *ChemPhysChem*, 2005, **6**, 1911–1916.

7 G. Henkelman, B. P. Uberuaga and H. Jónsson, *J. Chem. Phys.*, 2000, **113**, 9901–9904.

8 D. W. LYNCH and W. R. HUNTER, in *Handbook of Optical Constants of Solids*, Elsevier, 1998, p. 341.

9 M. W. Ribarsky, in *Handbook of Optical Constants of Solids*, Elsevier, 1997, pp. 795–804.

10 B. Choudhury and A. Choudhury, *Phys. E Low-dimensional Syst. Nanostructures*, 2014, **56**, 364–371.

11 J. M. Sanz, D. Ortiz, R. Alcaraz de la Osa, J. M. Saiz, F. González, A. S. Brown, M. Losurdo, H. O. Everitt and F. Moreno, *J. Phys. Chem. C*, 2013, **117**, 19606–19615.

12 X. Meng, T. Wang, L. Liu, S. Ouyang, P. Li, H. Hu, T. Kako, H. Iwai, A. Tanaka and J. Ye, *Angew. Chemie Int. Ed.*, 2014, **53**, 11478–11482.

13 H. Yin, Y. Kuwahara, K. Mori, H. Cheng, M. Wen and H. Yamashita, *J. Mater. Chem. A*, 2017, **5**, 8946–8953.

14 E. Prodan, *Science (80-.).*, 2003, **302**, 419–422.

15 C. Mao, H. Li, H. Gu, J. Wang, Y. Zou, G. Qi, J. Xu, F. Deng, W. Shen, J. Li, S. Liu, J. Zhao and L. Zhang, *Chem*, 2019, **5**, 2702–2717.

16 J. Yang, Y. Guo, R. Jiang, F. Qin, H. Zhang, W. Lu, J. Wang and J. C. Yu, *J. Am. Chem. Soc.*, 2018, **140**, 8497–8508.

17 H. Li, F. Qin, Z. Yang, X. Cui, J. Wang and L. Zhang, *J. Am. Chem. Soc.*, 2017, **139**, 3513–3521.
